# Acceleration of neuromelanin-sensitive MRI sequences in the substantia nigra using standard MRI options

**DOI:** 10.1007/s00234-022-03058-w

**Published:** 2022-09-28

**Authors:** Marieke van der Pluijm, Elon D. Wallert, Bram F. Coolen, Kaithlyn T. Tjong Tjin Joe, Lieuwe de Haan, Jan Booij, Elsmarieke van de Giessen

**Affiliations:** 1 Department of Radiology and Nuclear Medicine, Amsterdam UMC, University of Amsterdam, Amsterdam, The Netherlands; 2 Department of Psychiatry, Amsterdam UMC, University of Amsterdam, Amsterdam, The Netherlands; 3 Department of Biomedical Engineering & Physics, Amsterdam UMC, University of Amsterdam, Amsterdam, The Netherlands

**Keywords:** Neuromelanin, Substantia nigra, Acceleration, Reliability

## Abstract

**Purpose:**

Neuromelanin MRI (NM-MRI) is applied as a proxy measurement of dopaminergic functioning of the substantia nigra pars compacta (SN). To increase its clinical applicability, a fast and easily applicable NM-MRI sequence is needed. This study therefore compared accelerated NM-MRI sequences using standard available MRI options with a validated 2D gradient recalled echo NM-MRI sequence with off-resonance magnetization transfer (MT) pulse (2D-MToffRes).

**Methods:**

We used different combinations of compressed sense (CS) acceleration, repetition times (TR), and MT pulse to accelerate the validated 2D-MToffRes. In addition, we compared a recently introduced 3D sequence with the 2D-MToffRes.

**Results:**

Our results show that the 2D sequences perform best with good to excellent reliability. Only excellent intraclass correlation coefficients were found for the CS factor 2 sequences.

**Conclusion:**

We conclude that there are several reliable approaches to accelerate NM-MRI, in particular by using CS.

## Introduction

Neuromelanin MRI (NM-MRI) is becoming a key instrument for in-vivo visualization of dopaminergic functioning of the substantia nigra pars compacta (SN) and has potential for clinical application in disorders characterized by dopaminergic alterations such as Parkinson’s disease and schizophrenia [1]. Several NM-MRI sequences have been investigated, but most commonly used is the 2D gradient recalled echo NM-MRI sequence with off-resonance MT pulse (2D-MToffRes), as its contrast ratio (CR) has been validated with postmortem regional NM concentration and already successfully applied in clinical research [2]. An important drawback of the sequence is its scan duration of over 10 min. Customization of the standard MT pulse can reduce scan duration to 4–7 min; however, this is not readily applicable in clinical practice [3]. Instead, for a clinical protocol, it is essential to accelerate the scan using standard available MRI options. Most recently, a 3D sequence using on-resonance MT pulse with a scan duration of approximately 4 min has been introduced [4]. Advantages of 3D scanning are the potentially higher resolution for small structures such as the SN and a better contrast to noise ratio. This sequence has not been validated or compared to other sequences, yet. The aim of the current study is therefore to assess the performance in terms of CR in the SN of several accelerated NM-MRI sequences using standard available MRI options, including compressed sense (CS) and 3D scanning, and compare these with the validated 2D-MToffRes. Sequences with good to excellent reliability are considered useful alternatives.

## Methods

### Participants

This study was approved by the Medical Ethics Committee of the Amsterdam Medical Centre. All participants gave written informed consent prior to the scan after the procedure had been fully explained. Nine healthy participants (aged 26.2 ± 3.3 years; 5 males) were included in de CS protocol and 10 healthy participants (aged 26.3 ± 6.1 years; 2 males) in the 3D protocol. All participants were aged between 18 and 40 years. Prior to inclusion, participants were screened by means of an interview and excluded if they had a neurological or psychiatric disorder or had any MRI contraindication.

### Image acquisition

All MR data were acquired using a 3 Tesla Ingenia MRI system (Philips, Best, The Netherlands) with a 32 channel SENSE head coil. For slice placement and registration, high-resolution structural T1-weighted volumetric images were acquired (TE/TR = 4.1/9.0 ms; 189 slices; FOV = 284 × 284 × 170 mm; voxel size = 0.9 × 0.9 × 0.9 mm, FA = 8°). Two protocols with different NM-MRI sequences were acquired (Table 1). In the CS protocol, we used CS factors 2 and 3 and adjusted repetition time (TR) to assure most efficient scanning (i.e., using only one slice package instead of three in the original 2D MToffRes). In the 3D protocol, we acquired 3D sequences with different FA and one 2D sequence without MT pulse preparation. For all NM-MRI scans, the axial slice orientation was the anterior commissure to posterior commissure line. To calculate the CR in the SN, we applied both manual and standardized analysis methods with crus cerebri (CC) as reference region.

**Table 1:** Scan parameters

Parameter	TE (ms)	TR (ms)	FA°	Slices	Slice gap	Spatial resolution (mm)	FOV (mm)	NSA	Accel. factor	MT offset (Hz)	MT dur. (ms)	Acq. time (min)
CS protocol												
2D-MToffRes	3.9	260	40	8	0.25	0.39 × 0.39 × 2.5	162 × 199	2	-	1200	15.6	13:20
2D-noMTRes	3.9	260	40	8	0.25	0.39 × 0.39 × 2.5	162 × 199	2	-	-	-	04:26
2D-CS2	3.9	260	40	8	0.25	0.39 × 0.39 × 2.5	162 × 199	2	CS = 2	1200	15.6	06:42
2D-CS3	3.9	260	40	8	0.25	0.39 × 0.39 × 2.5	162 × 199	2	CS = 3	1200	15.6	04:28
2D-TRad	3.9	633	40	8	0.25	0.39 × 0.39 × 2.5	162 × 199	2	-	1200	15.6	10:47
2D-TRad-CS2	3.9	633	40	8	0.25	0.39 × 0.39 × 2.5	162 × 199	2	CS = 2	1200	15.6	05:25
2D-TRad-CS3	3.9	633	40	8	0.25	0.39 × 0.39 × 2.5	162 × 199	2	CS = 3	1200	15.6	03:36
3D protocol												
2D-MToffRes	3.9	260	40	8	0.25	0.39 × 0.39 × 2.5	162 × 199	2	-	1200	15.6	13:20
2D-MTonRes	3.9	260	40	8	0.25	0.39 × 0.39 × 2.5	162 × 199	2	-	0	8.5	08:54
3D-FA12	7.5	62	12	48	-	0.67 × 0.67 × 1.34	256 × 192	1	S = 2	0	8.5	04:03
3D-FA15	7.5	62	15	48	-	0.67 × 0.67 × 1.34	256 × 192	1	S = 2	0	8.5	04:03
3D-FA25	7.5	62	25	48	-	0.67 × 0.67 × 1.34	256 × 192	1	S = 2	0	8.5	04:03

The CS protocol consisted of (1) the original 2D NM-MRI with off-resonance MT pulse (2D-MToffRes); (2) the original 2D NM-MRI without MT pulse (2D-MTno); (3/4) the 2D-MToffRes with CS factor 2 and 3 (2D-CS2 and 2D-CS3, respectively); (5/6/7) the 2D-MToffRes with an adjusted TR of 633 ms (2D-TRad) and with CS factor 2 and 3 (2D-TRad-CS2 and 2D-TRad-CS3, respectively). The 3D protocol consisted of (1) the 2D-MToffRes; (2) the original 2D NM-MRI with on-resonance MT pulse (2D-MTonRes); (3/4/5) a 3D NM-MRI scan with on-resonance MT pulse and a flip angle of 12, 15, and 25 (3D-FA12, 3D-FA15, and 3D-FA25, respectively). *TE*, echo time; *TR*, repetition time; *FA*, flip angle; *FOV*, field of view; *NSA*, number of signal averages; *Accel. factor*, acceleration factor; *MT*, magnetization transfer; *Acq. time*, acquisition time; *CS*, compressed sense; *S*, sense

### Manual analysis

The SN was manually segmented on the three consecutive slices and six consecutive slices, for the 2D and 3D respectively, with the highest voxel intensity using ITK-Snap (v. 3.6.0). The CC was segmented as reference region and consisted of six default circles three on each side of the SN. Segmentation was performed by K. T. T. J., who was trained and performed over a 100 segmentations of the SN on NM-MRI prior to this research. The CR ([S_SN_ − S_CC_]/S_CC_) was calculated as described previously [2, 5], where S_SN_ and S_CC_ illustrate the mean signal intensities of the SN and CC, respectively. In addition, we calculated the contrast to noise ratio (CNR = [S_SN_ − S_CC_]/SD_CC_) as a measure for image quality with the standard deviation of the CC (SD_cc_) representing the noise [6].

### Standardized analysis

In addition, the NM-MRI scans were analyzed using a pipeline from a previous study [3]. All NM-MRI data were normalized to MNI standard space and spatially smoothed with a 1-mm full-width-at-half-maximum Gaussian kernel using ANTS software. Template masks of the SN and CC were created by manual tracing with ITK-Snap on a standardized average image of all 2D-MToffRes scans. The CR was calculated at each voxel in the SN mask using the CC as reference region. The mean CR per participant was acquired by averaging the CR values of all voxels in the SN mask that had a non-negative value.

### Statistical analyses

Statistical analyses were performed in SPSS [7]. Sequences were compared with the validated original 2D-MToffRes using the intraclass correlation coefficients (ICC) from a mixed consistency model and Pearson correlation coefficients. ICC values > 0.75 are considered good and > 0.90 excellent [8].

## Results

**Fig. 1:**
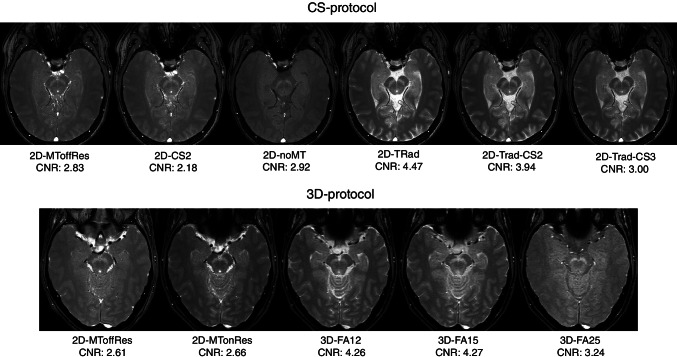
Overview of the neuromelanin MRI scans. An example of the neuromelanin MRI sequences of one participant of the CS protocol and one participant of the 3D protocol, with the mean contrast to noise ratio (CNR) of all participants per scan. MT, magnetization transfer; Res, resonance; CS, compressed sense; TRad, repetition time adjusted; FA, flip angle

An overview of the NM-MRI scans are depicted in Fig. 1. The scans demonstrated a CNR between 2.18 and 4.47, with the highest CNR for the 2D sequence with a TR of 633 ms and 3D sequences. CR was lowest for the 3D sequence with a FA of 25 and highest for the 2D sequence with a TR of 633 ms (Table 2). Only excellent ICCs were found for the sequences with CS factor 2, with a TR of 260 ms and 633 ms (Table 2). The manual analysis performed worse than the standardized analysis.

**Table 2:** Mean contrast ratio and reliability of the different sequences

	Manual analysis						Standardized analysis					
	Mean	SD	*R*	*p*-value	ICC	*p*-value	Mean	SD	*R*	*p*-value	ICC	*p*-value
CS protocol												
2D-MToffRes	21.81	1.83					16.60	1.82				
2D-noMT	20.93	1.55	0.79	0.06	0.79	0.02	16.72	1.31	0.83	0.04	0.79	0.02
2D-CS2	20.37	1.51	0.55	0.12	0.54	0.05	15.01	1.96	0.91	0.00	0.91	0.00
2D-TRad	27.29	2.93	0.91	0.00	0.82	0.00	20.05	2.27	0.86	0.00	0.84	0.00
2D-TRad-CS2	26.21	3.11	0.87	0.00	0.76	0.01	19.09	2.02	0.93	0.00	0.93	< 0.001
2D-TRad-CS3	26.21	3.18	0.89	0.00	0.79	0.01	19.30	1.74	0.87	0.01	0.86	0.00
3D-protocol												
2D-MToffRes	19.33	2.06					14.94	1.42				
2D-MTonRes	24.51	2.37	0.91	0.00	0.90	0.00	18.71	2.28	0.88	0.00	0.69	0.01
3D-FA12	24.78	3.97	0.80	0.01	0.66	0.01	17.24	5.15	0.80	0.01	0.41	0.11
3D-FA15	22.56	3.58	0.87	0.00	0.75	0.00	13.23	3.25	0.77	0.01	0.56	0.04
3D-FA25	15.95	1.91	0.84	0.00	0.84	< 0.001	9.64	2.27	0.66	0.04	0.60	0.03

*TRad*, repetition time adjusted; *FA*, flip angle; *MT*, magnetization transfer; *Res*, resonance; *CS*, compressed sense; *SD*, standard deviation; *R*, Pearson’s correlation coefficient; *ICC*, intraclass correlation coefficient (mixed consistency model)

## Discussion

This study demonstrates that several directly applicable strategies to accelerate NM-MRI show good to excellent reliability compared to the validated 2D-MToffRes sequence. The 2D sequence with a TR of 633 ms and 3D sequences demonstrate the highest CNR, as expected for the 3D sequences [4]. However, in terms of reliability, the 3D sequences perform worse than the 2D sequences. The 2D sequences with CS most reliably correspond to the validated 2D-MToffRes, especially using the standardized analysis protocol. The sequences with CS factor 2 show excellent ICCs, which is unaffected by increasing TR.

We used CS to accelerate the sequences as CS does not affect the MT and T1-shortening effects, but instead undersamples *k-*space to reduce scan time [9]. The paramagnetic neuromelanin-iron complexes in combination with the high water content of neuromelanin compared to the surrounding tissues lead to the T1-shortening and MT effects which are thought to underlie the contrast [10]. We assessed the CS sequences also with adjusted TR. Using a TR of 260 ms on our scanner resulted in separating the slices in 3 packages. This was less time efficient, because of the incorporated waiting time due to specific absorption rate limits. Adjusting the TR to 633 ms made it possible to fit all slices in one package, decreasing scan duration. In addition, more slices in one package increases the multi-slice MT effect and thereby the CR [11]. Higher CR could result in easier manual tracing of the SN and may thereby explain the better performance of the manual analysis [5]. It should be noted though that the 3D sequences with higher CR and CNR show lower ICCs. This underlines that higher CR and CNR are not necessarily more reliable and stresses the importance of comparing optimized sequences to a validated sequence or post mortem data.

The 3D sequences show a relatively high CNR and moderate to good ICCs using the manual analysis; however, they fail to reach good ICCs using the standardized analysis. The manual analysis is biased though by intra-rater differences and the circularity of acquiring the CR in a mask based on the highest contrast [5]. Therefore, the results of the standardized analysis are an important indication that the 3D sequences are less reliable for semi-quantification than the 2D sequences. This finding is in line with the results of a meta-analysis on findings in Parkinson’s disease, showing that studies using 2D sequences report a slightly better diagnostic performance than studies using a 3D sequence [12]. We should mention though that the standardized analysis is validated for the 2D sequences and might be less accurate for the 3D sequences [3]. Advantages of the 3D sequences are the short scan duration and high resolution. They may be useful for anatomical localization of the SN. However, for semi-quantitative purposes, the 2D sequences with CS appear to be most reliable.

We also adjusted the MT effects by changing the MT pulse to the on-resonance MT pulse and by omitting the MT pulse. The on-resonance MT pulse is more time efficient than the off-resonance MT sequence which requires a longer TR to avoid too high specific absorption rates. Adjusting the MT pulse did not appear to markedly affect the reliability. It should be noted that the 2D sequence without MT pulse was scanned in one package and thereby the increased multi-slice MT effect could have compensated the effect of the off-resonance MT pulse.

Interestingly, our results show some difference in CR for the validated 2D-MToffRes sequence between the two protocols. It should be noted that the CS protocol and 3D protocol consisted of different participant samples and, albeit scanned on the same scanner, the protocols were scanned more than a year apart during which two scanner software updates occurred. The difference is not likely related to reproducibility issues since several NM-MRI 2D sequences have demonstrated a good to excellent reproducibility [3, 13], including the validated 2D-MToffRes sequence used in this study with a test–retest variability below 6% [5].

Finally, it will be essential to further validate the accelerated sequences in patient samples. Our samples consisted of small and homogenous groups (e.g., similar age), making the statistical results more prone to limited variation in data points.

To conclude, there are several reliable approaches to accelerate NM-MRI. CS or similar acceleration techniques appear to be most suitable for semi-quantitative purposes.
